# miR-142-5p promotes TSCM differentiation and suppresses progressive T-cell maturation via targeting PRKCB

**DOI:** 10.3389/fimmu.2026.1807053

**Published:** 2026-05-18

**Authors:** Hongqiong Wang, Shengfang Xia, Jia Chen, Huishan Zhong, Xianpei Zeng, Zitao Lu, Wenfeng Zhang, Fenglin Wu

**Affiliations:** 1 School of Life Sciences, Guangdong Pharmaceutical University, Guangzhou, Guangdong, China; 2 Department of Science and Education Division, Public Health Clinical Center of Chengdu, Chengdu, Sichuan, China

**Keywords:** immunotherapies, miR-142-5p, PRKCB, progressive differentiation, T cells

## Abstract

**Introduction:**

Adoptive cellular immunotherapy (ACT) such as CAR‑T therapy holds promise for cancer treatment. However, genetically engineered T cells often undergo terminal differentiation during ex vivo expansion, which limits their persistence and antitumor efficacy *in vivo*. Early‑differentiated T‑cell subsets exhibit better survival and proliferative capacity after infusion. In our previous work, we isolated four T‑cell subsets at different differentiation stages: naïve T cells (T_N_), stem cell‑like memory T cells (TSCM), central memory T cells (T_CM_), and effector memory T cells (T_EM_), and obtained their miRNA expression profiles via high‑throughput sequencing. In the present study, we found that hsa‑miR‑142‑5p is highly expressed in TSCM cells and gradually decreases during T‑cell differentiation.

**Methods:**

Bioinformatics analysis suggested that miR‑142‑5p target genes are involved in transcription regulation. We identified four candidate targets through reverse enrichment analysis. Dual‑luciferase reporter assays were used to validate direct targets. Functional studies were then performed in T cells overexpressing miR‑142‑5p to assess expression of differentiation‑associated and effector‑related genes, surface markers (CCR7, CD62L, CD95), cell subset proportions (T_N_, T_SCM_, T_EM_/T_EFF_), proliferation, apoptosis, and cytokine secretion (TNF‑α, IFN‑γ).

**Results:**

Dual‑luciferase reporter assays confirmed PRKCB as a direct target of miR‑142‑5p, and miR‑142‑5p suppressed PRKCB expression in T cells. miR‑142‑5p overexpression upregulated early differentiation‑associated genes (LEF1, CD62L, CCR7) and the anti‑apoptotic gene BCL2, while downregulating late differentiation‑associated genes (KLRG1, EOMES, PDCD1) and effector function‑related genes (GZMB, PRF1). Consistently, it enhanced early differentiation markers (CCR7, CD62L) and reduced the late marker CD95. It also increased T_N_ and T_SCM_ proportions while decreasing T_EM_/T_EFF_ cells. Additionally, miR‑142‑5p promoted T‑cell proliferation, reduced apoptosis, and suppressed TNF‑α and IFN‑γ secretion.

**Discussion:**

In summary, miR‑142‑5p inhibits progressive T‑cell differentiation by directly targeting PRKCB, helping maintain an early‑differentiated phenotype. This offers a potential strategy to improve the persistence and efficacy of adoptive T‑cell‑based immunotherapies.

## 1 Introduction

Adoptive cellular therapy (ACT), exemplified by chimeric antigen receptor T-cell (CAR-T) technology, has provided an innovative therapeutic option for cancer treatment. To date, multiple CAR-T cell products have been approved for hematologic malignancies. These include diffuse large B-cell lymphoma, acute lymphoblastic leukemia, and multiple myeloma (1). CAR-T therapies are also being evaluated in clinical trials for various solid tumors, such as gastrointestinal cancers and other refractory malignancies.

The antitumor efficacy of ACT largely depends on three key factors: expansion, long-term persistence, and continued antitumor activity of adoptively transferred cells (2). Current ACT strategies — taking CAR-T and TCR-T therapies as examples. They typically involve the *in vitro* activation and genetic modification of autologous peripheral blood T cells. The process generates sufficient numbers of tumor-reactive T cells for infusion. However, these engineered T cells predominantly exhibit an effector T-cell phenotype. While effector T cells possess potent cytotoxic activity, they are prone to terminal differentiation during *in vitro* expansion. This leads to functional exhaustion and reduced persistence, ultimately limiting their therapeutic efficacy *in vivo*.

Accumulating clinical studies indicate that T cells at earlier stages of differentiation exhibit superior expansion and long-term survival capacity. These include naïve T cells and memory T cells (3). Among these subsets, stem cell-like memory T cells (T_SCM_) are particularly notable. They retain self-renewal ability and can further differentiate into effector progeny that elicit antitumor responses. Accordingly, extensive efforts have been devoted to enriching or generating early-differentiated T-cell populations. These strategies include optimization of *in vitro* culture systems, modulation of CAR signaling strength, intervention in T-cell differentiation pathways, regulation of epigenetic states, and targeting of cellular metabolic programs.

MicroRNAs (miRNAs) have emerged as critical post-transcriptional regulators of T cell differentiation (4). These small noncoding RNAs, approximately 22 nucleotides in length, regulate gene expression by binding to the 3′ untranslated regions (3′-UTRs) of target mRNAs. During T cell differentiation, miRNA expression undergoes dynamic changes and forms complex regulatory networks. These networks play essential roles in T cell activation and fate determination. In CD8^+^ T cells, for example, activation triggers extensive remodeling of the miRNA expression landscape. Several miRNAs—including miR-15b, miR-142-3p, miR-150, and miR-16—are highly expressed in naïve T cells. In contrast, upregulation of the miR-17~92 cluster is closely associated with effector T cell activation and expansion, followed by a gradual decline during memory development. Additionally, both miR-150 and miR-143 promote memory T cell differentiation. The miR-15/16 family also plays a critical role in generating long-lived memory T cells through coordinated regulation of multiple target genes.

In our previous work, we isolated four T-cell subsets representing distinct stages of progressive differentiation: naïve T cells (T_N_), stem cell-like memory T cells (T_SCM_), central memory T cells (T_CM_), and effector memory T cells (T_EM_). We generated their miRNA expression profiles using high-throughput sequencing (5). In the present study, we performed differential expression analysis of miRNA profiles across these four subsets. We focused particularly on T_SCM_ cells, which reside at the earliest stage of antigen-stimulated progressive differentiation and possess stem cell-like properties. Through this analysis, we identified hsa-miR-142-5p as being highly expressed in T_SCM_.

## 2 Materials and methods

### 2.1 Identification of differentially expressed miRNAs

You may insert up to 5 heading levels into your manuscript as can be seen in “Styles” tab of this template. These formatting styles are meant as a guide, as long as the heading levels are clear, Frontiers style will be applied during typesetting. Based on the miRNA expression profiles obtained in our previous study, the raw data were first subjected to TPM normalization. To investigate the dynamic changes of miRNAs during progressive T-cell differentiation, the four T-cell subsets (T_N_, T_SCM_, T_CM_, T_EM_) were divided into three comparison groups. Pairwise comparisons were performed between consecutive stages: T_SCM_ vs. T_N_, T_SCM_ vs. T_CM_, and T_SCM_ vs. T_EM_, to identify differentially expressed miRNAs (DEmiRNAs) at each transitional step.

Differential expression analysis was conducted using the DESeq2 package in R (6). miRNAs were considered significantly differentially expressed if they met the following criteria: absolute Fold Change (FC) ≥ 2 (|log_2_ FC| ≥ 1) with a false discovery rate (FDR) adjusted p-value (p. adjusted) ≤ 0.05. The p-values were corrected for multiple testing using the Benjamini-Hochberg method (7). Finally, a clustered heatmap of the identified DEmiRNAs was generated using the pheatmap package (version 1.0.12).

### 2.2 miRNA target gene prediction and regulatory network visualization

To elucidate the potential functions of differentially expressed miRNAs (DEmiRNAs) across T-cell subsets, we performed systematic target gene prediction. Two databases were used: miRWalk 2.0 (8) and StarBase (9). StarBase provides Argonaute CLIP-seq validated binding sites (10). The predicted target genes were then subjected to functional enrichment analysis. Gene Ontology (GO) enrichment and Kyoto Encyclopedia of Genes and Genomes (KEGG) pathway analysis were performed using the R package clusterProfiler (version 4.0). Local annotation databases were used as references. Significant enrichment was defined as a false discovery rate (FDR)-corrected p-value < 0.05. A miRNA-target gene regulatory network was constructed and visualized. This was done using the iRegulon plugin (version 1.3) within the Cytoscape platform (version 3.10.4).

### 2.3 T cell activation

Peripheral blood mononuclear cells (PBMCs) were resuspended at a density of 1×10^6^ cells/mL in RPMI-1640 basic medium (Gibco™, USA) supplemented with 10% fetal bovine serum (FBS) (BioSharp, China). For *in vitro* stimulation, cells were incubated for 72 hours with 2 μg/mL anti-CD3 antibody (BioLegend, USA), 1 μg/mL anti-CD28 antibody (BioLegend, USA), and 300 U/mL recombinant human IL-2 (PeproTech, USA). Subsequently, T cells were transfected using Lipofectamine™ 3000 transfection reagent (Invitrogen™, USA) with either miR-142-5p mimic, mimic negative control (NC), miR-142-5p inhibitor, or inhibitor NC (Ribobio, China), strictly following the manufacturer’s protocol.

### 2.4 Dual-luciferase reporter assay for miRNA-142-5p target validation

DNA fragments encompassing approximately 200 bp upstream and downstream of the predicted miR-142-5p binding sites within the 3′ untranslated regions (3′-UTRs) of candidate genes (PRKCB, PURA, ANKRD17, CAMK2K) were cloned into the psiCHECK-2 reporter vector (11). Mutant reporter plasmids were generated via site-directed mutagenesis of the wild-type (WT) constructs based on PCR. Additionally, to create a miRNA sensor (miRNA pos) plasmid, the reverse complementary sequence of miR-142-5p was cloned into the psiCHECK-2 vector. For transfection, 293T cells were seeded in 6-well plates at 1 × 10^6^ cells/well and cultured for 24 hours. Following the Lipofectamine™ 3000 protocol, cells were co-transfected with either the WT or mutant reporter plasmid (or the miRNA pos/empty vector control) alongside the miR-142-5p mimic or mimic NC. After 48 hours, cells were harvested, and luciferase activity was measured using a Dual-Luciferase Reporter Assay System (Yeasen, Shanghai, China).

### 2.5 RNA extraction and quantitative real-time PCR

Total RNA was extracted from cells using the EZ-press RNA Purification Kit (EZBioscience). Complementary DNA (cDNA) was synthesized using the Color Reverse Transcription Kit (EZBioscience). Quantitative PCR was performed using 2× SYBR Green qPCR Master Mix (EZBioscience) on a LightCycler^®^ 480 Instrument II (Roche). The relative expression levels of target genes were calculated using the 2^−^ΔΔCt method. Primer sequences are listed in **Supplementary Table 1**.

### 2.6 Western blot analysis

T cell protein lysates were prepared using RIPA Buffer (Beyotime Biotechnology). Protein concentration was quantified using a BCA Protein Assay Kit (Beyotime). Proteins were separated by electrophoresis on 8%-20% SDS-polyacrylamide gels at a constant voltage of 150 V and subsequently transferred onto polyvinylidene difluoride (PVDF) membranes (Biosharp). Membranes were blocked with 5% non-fat milk at room temperature for 2 hours, followed by overnight incubation at 4 °C with primary antibodies against GAPDH (ProteinTech, 1:5000 dilution) and PRKCB (Abcam, 1:5000 dilution). After washing, membranes were incubated with HRP-conjugated goat anti-mouse secondary antibody (Cell Signaling Technology, 1:5000 dilution) for 1 hour at room temperature. Immunoreactive bands were visualized using ECL substrate (New Cell & Molecular Biotech) and detected.

### 2.7 Flow cytometry

**Figure 1:**
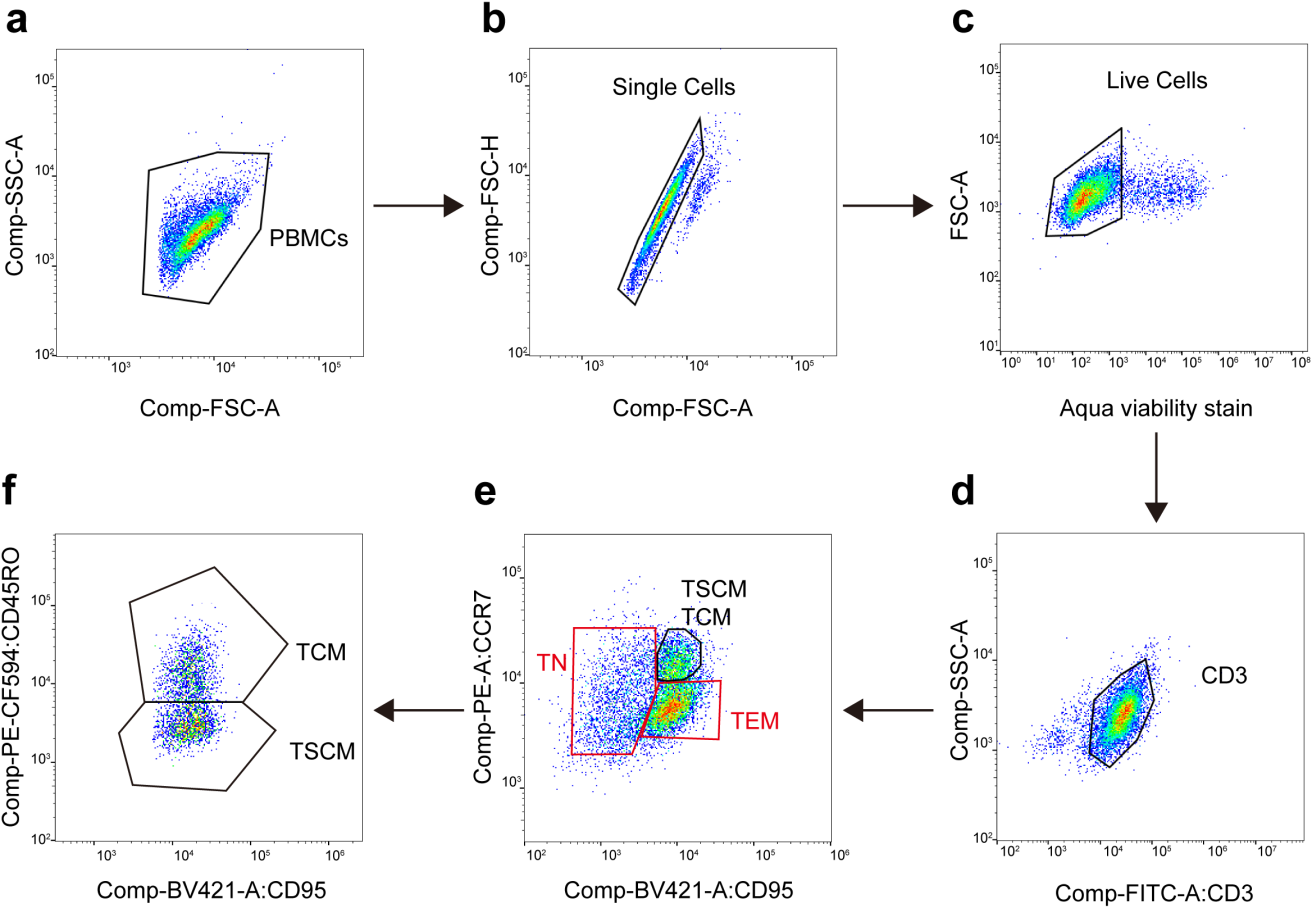
Flow cytometry gating strategy for T-cell subset identification. **(a-d)** Lymphocytes were first gated based on forward and side scatter (FSC-A vs SSC-A). Doublets were excluded using FSC-H vs FSC-A. Live cells were selected as Aqua viability stain-negative. CD3^+^ T cells were then identified. **(e)** Within the CD3^+^ T cell population, cells were plotted for CCR7 vs CD95. Three distinct populations were defined: CCR7^+^ CD95^−^ cells were identified as naïve T cells (T_N_); CCR7^+^ CD95^+^ cells contained both stem cell memory T (T_SCM_) and central memory T (T_CM_) subsets; and CCR7^−^ CD95^+^ cells were identified as effector memory T (T_EM_) cells. **(f)** Within the CCR7^+^ CD95^+^ population, cells were further analyzed for CD45RO expression. T_SCM_ were defined as CD45RO^−^, while T_CM_ were defined as CD45RO^+^.

PBMCs were harvested for analysis 5 days after transfection with the miR-142-5p mimic or inhibitor. For immunophenotyping, cells were stained with the following panel of fluorescently conjugated antibodies for 30 minutes at 4 °C in the dark: anti-CD3-FITC, anti-CCR7-PE, anti-CD45RO-PE-CF594, and anti-CD95-BV421 (Thermo Fisher Scientific, USA) (12). The gating strategy for T-cell subset identification is shown in **Figure 1**. Briefly, lymphocytes were first gated based on forward and side scatter (FSC-A vs SSC-A). Doublets were excluded using FSC-H vs FSC-A. Live cells were then selected as Aqua viability stain-negative cells. Within the live cell population, CD3^+^ T cells were identified. Among CD3^+^ T cells, the following subsets were defined based on CCR7, CD45RO, and CD95 expression:

Naïve T cells (T_N_): CCR7^+^ CD45RO^−^ CD95^−^

Stem cell memory T cells (T_SCM_): CCR7^+^ CD45RO^−^ CD95^+^

Central memory T cells (T_CM_): CCR7^+^ CD45RO^+^ CD95^+^

Effector memory/effector T cells (T_EM_/T_EFF_): CCR7^−^ CD45RO^+^ CD95^+^

### 2.8 Apoptosis and proliferation assays

For proliferation analysis, cell suspensions from different treatment groups were seeded into 96-well plates at 5×10³ cells/well. Cell Counting Kit-8 (CCK-8; YEASEN) reagent was added (10 μL/well), and plates were incubated at 37 °C with 5% CO_2_ for 4 hours. Absorbance was measured at 450 nm using a microplate reader (Varioskan Flash, Thermo Scientific). Apoptosis was assessed using an Annexin V-FITC/PI Apoptosis Detection Kit (MultiSciences) on cells cultured for 5 days post-transfection in medium containing 50 U/mL IL-2. The proportions of viable, apoptotic, and dead cells were analyzed using FlowJo software (version 10).

### 2.9 Enzyme-linked immunosorbent assay

Cell culture supernatants were collected and centrifuged at room temperature. Concentrations of secreted IFN-γ and TNF-α were measured using a Human IFN-γ ELISA Kit and a Human TNF-α ELISA Kit (MultiSciences), respectively, according to the manufacturer’s instructions.

### 2.10 Statistical analysis

All experiments were performed independently at least three times. Quantitative data are presented as mean ± standard deviation (SD). Statistical analysis was performed using GraphPad Prism (version 8.0). Comparisons between two groups were analyzed using Student’s t-test, while comparisons among multiple groups were performed using one-way analysis of variance (ANOVA). A p-value < 0.05 was considered statistically significant.

## 3 Result

### 3.1 Screening of differentially expressed miRNAs in the T memory stem cells

Based on antigen exposure and differentiation status following antigen-induced activation, T cells can be classified into multiple subsets, including naïve T cells (T_N_), stem cell–like memory T cells (T_SCM_), central memory T cells (T_CM_), effector memory T cells (T_EM_), and effector T cells (T_EFF_). In our previous study, four distinct T-cell subsets were isolated from peripheral blood samples using flow cytometry sorting. These subsets included naïve T cells (T_N_), stem cell-like memory T cells (T_SCM_), central memory T cells (T_CM_), and effector memory T cells (T_EM_). Blood samples were collected from three healthy donors, yielding a total of 12 samples. Total RNA was then extracted from each sample. Owing to the low frequency and limited cell numbers of T_SCM_ within the total T-cell population, the RNA concentration of the T_SCM_ sample derived from Donor 2 did not meet the minimum requirements for sequencing. Consequently, high-throughput sequencing was performed on the remaining 11 samples using the Illumina NovaSeq 6000 platform. Following raw data filtering, quality control, and sequence alignment, miRNA expression profiles for the four T-cell subsets were generated.

**Figure 2:**
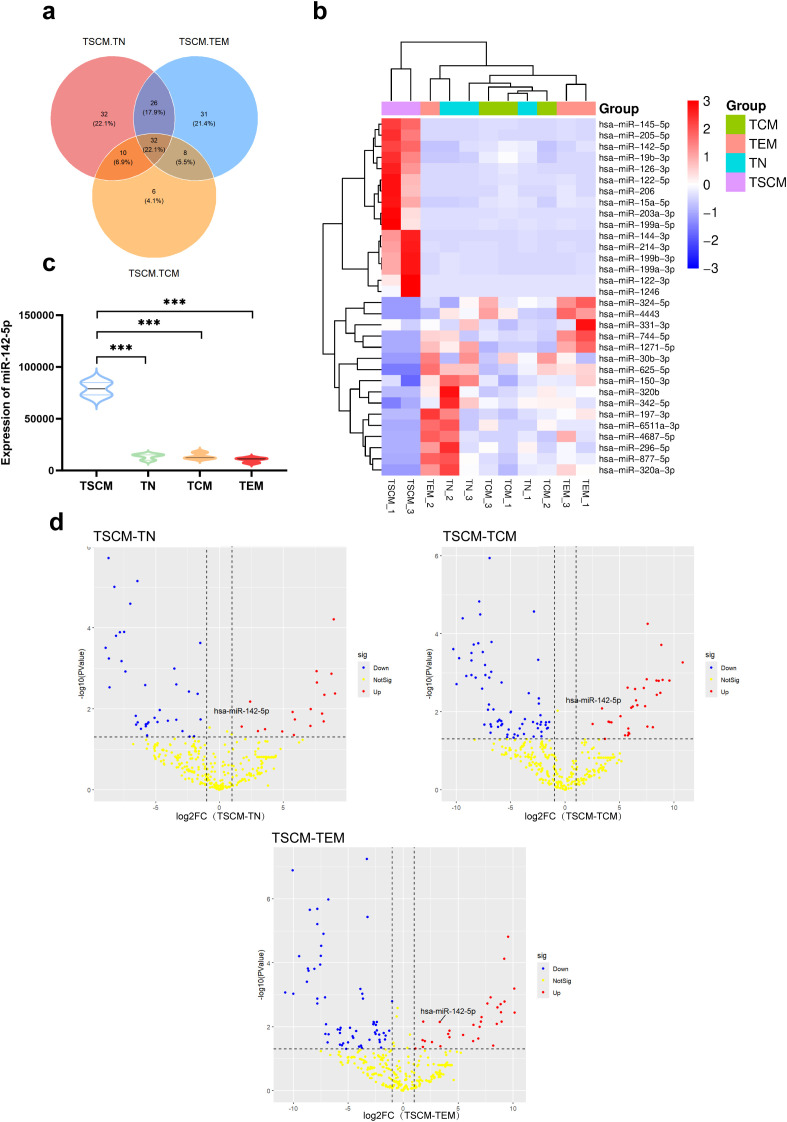
Differential miRNA expression in T_SCM_ compared with T_N_, T_CM_, and T_EM_ subsets. **(a)** The intersection of differentially expressed miRNAs identified from the three pairwise comparisons (T_SCM_ vs. T_N_, T_SCM_ vs. T_CM_, and T_SCM_ vs. T_EM_) was determined, yielding 32 miRNAs with significant differential expression in T_SCM_. Pink indicates miRNAs differentially expressed in T_SCM_ vs. T_N_, blue indicates those in T_SCM_ vs. T_EM_, and orange indicates those in T_SCM_ vs. T_CM_. **(b)** A clustered heatmap was generated based on the 32 differentially expressed miRNAs in T_SCM_. Each row represents an individual miRNA, and each column represents a single sample. Color intensity indicates miRNA expression levels (red: high expression; blue: low expression). A dendrogram illustrating miRNA clustering is shown on the left, miRNA names are listed on the right, and sample names are displayed at the bottom. **(c)** The expression of miR-142-5p across different T-cell subsets was validated by quantitative real-time polymerase chain reaction (qPCR). U6 was used as the internal reference, and cDNAs derived from the four T-cell subsets served as templates, with three biological replicates and three technical replicates included for each group. After determination of cycle threshold (Ct) values, relative expression levels were calculated using the 2^−^ΔΔCt method and subjected to statistical analysis. As shown, miR-142-5p was significantly upregulated in T_SCM_ compared with T_N_, T_CM_, and T_EM_ subsets (P < 0.001), consistent with the miRNAs sequencing results. Data are presented as mean ± standard error of the mean (SEM), n = 3. Statistical significance was determined using one-way analysis of variance (ANOVA). ***P < 0.001. **(d)** Volcano plots were constructed for the differentially expressed miRNAs in each of the three comparison groups (T_SCM_ vs. T_N_, T_SCM_ vs. T_CM_, and T_SCM_ vs. T_EM_). Screening thresholds were set as P < 0.05 and |log2FC| ≥ 1. The y-axis represents −log10(P values), with color gradients corresponding to the magnitude of −log10(P values) (red, higher significance; blue, lower significance), while the x-axis represents log2 fold change (log2FC). miR-142-5p is highlighted in each plot.

To identify miRNAs with differential expression in the T_SCM_ subset, differential expression analysis of miRNAs was performed using the R programming language with the DESeq2 package. The comparison groups were defined as T_SCM_ vs. T_N_, T_SCM_ vs. T_CM_, and T_SCM_ vs. T_EM_, with the latter subset in each comparison serving as the control. Differentially expressed miRNAs between T_SCM_ and each of the other subsets (T_N_, T_CM_, and T_EM_) were identified separately, and the intersection of these miRNAs was subsequently obtained (**Figure 2a**).

This screening identified 32 miRNAs with significantly differential expression in T_SCM_ subset, among which the highly expressed miRNAs included hsa-miR-744-5p, hsa-miR-1271-5p, and hsa-miR-320b. A clustered heatmap of these 32 differentially expressed miRNAs was generated using the Complex Heatmap package (**Figure 2b**).

Ranking the differentially expressed miRNAs by P value revealed that hsa-miR-142-5p was the most statistically significant. It also displayed the highest expression level in T_SCM_ compared to other miRNAs.

Quantitative real-time PCR (qPCR) was then performed to validate miR-142-5p expression across all T-cell subsets (**Figure 2c**). The qPCR results were consistent with the miRNA sequencing data, further confirming that miR-142-5p was most highly expressed in T_SCM_. Finally, volcano plots were constructed based on the differentially expressed miRNAs from the three comparison groups (T_SCM_ vs. T_N_, T_SCM_ vs. T_CM_, and T_SCM_ vs. T_EM_) (**Figure 2d**). Across all three comparisons, miR-142-5p exhibited an absolute log2 fold change (|log2FC|) ≥ 1 and a −log10(P value) ≥ 2, with markedly higher expression in T_SCM_. Accordingly, this study focused on elucidating the role of hsa-miR-142-5p in T-cell differentiation.

### 3.2 miR-142-5p potentially regulates T-cell differentiation and proliferation

According to the progressive differentiation model of T cells, naïve T cells undergo antigen stimulation and differentiate along the T_N_–T_SCM_–T_CM_–T_EM_–T_EFF_ trajectory. At distinct stages of differentiation, T cells exhibit distinct miRNA expression profiles, and miRNAs may influence the differentiation process by regulating their target genes. Based on differential miRNA expression analysis, miR-142-5p was expressed at low levels in T_N_ cells and at high levels in T_SCM_ cells, and its expression was subsequently downregulated as T cells underwent progressive differentiation, suggesting that miR-142-5p expression may be associated with the progressive differentiation of T cells. To further elucidate the role of miR-142-5p in T-cell differentiation, we analyzed the 32 miRNAs exhibiting significant differential expression in the T_SCM_ subsets and performed target gene prediction for the 16 miRNAs significantly upregulated in T_SCM_ cells.

**Figure 3:**
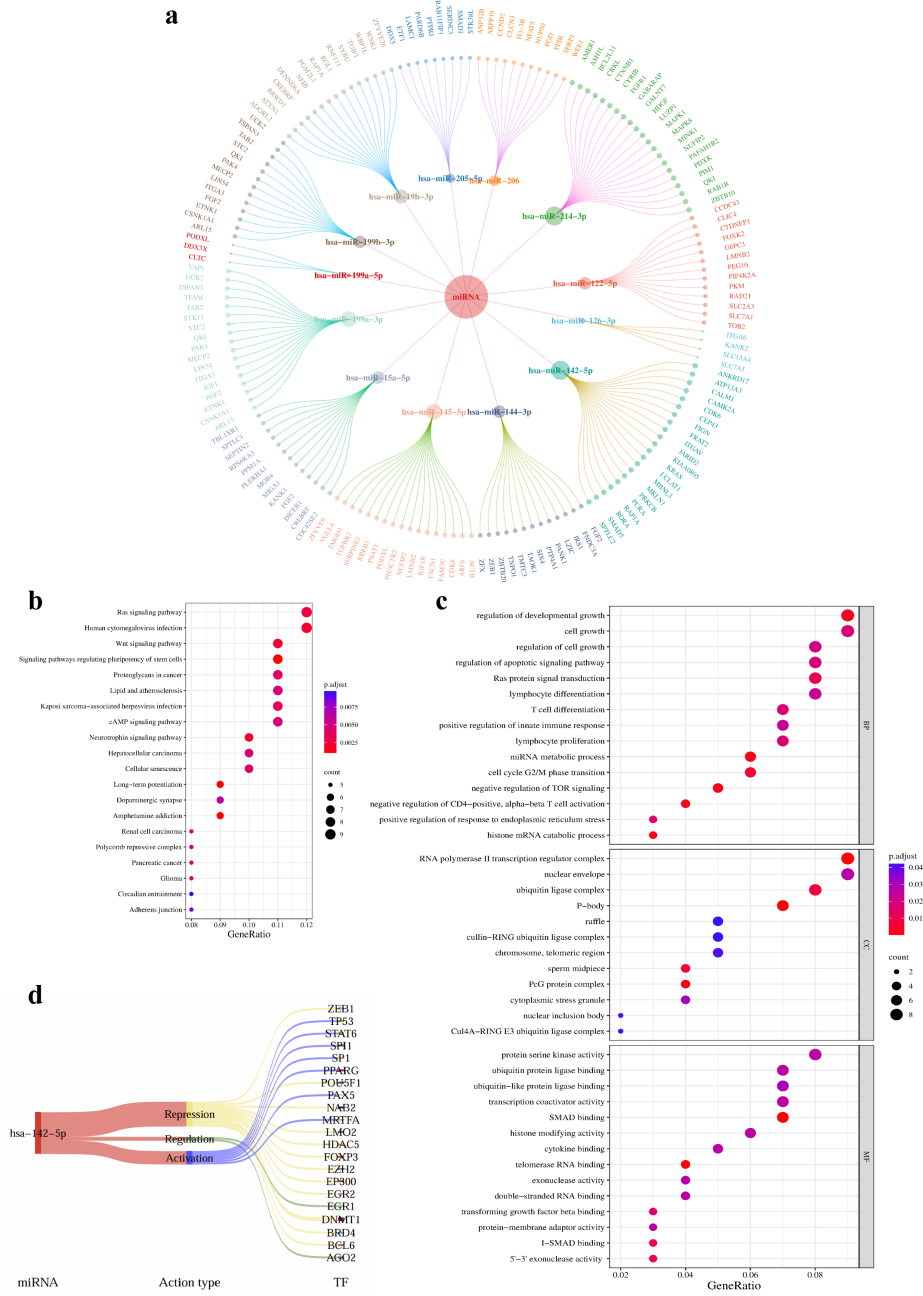
Analysis of miR-142-5p–regulated target genes and their functional enrichment. **(a)** A miRNA–target gene regulatory network was constructed. The inner circular nodes represent miRNAs significantly upregulated in T_SCM_, while the outer circular nodes represent their corresponding predicted target genes. **(b)** Kyoto Encyclopedia of Genes and Genomes (KEGG) pathway enrichment analysis was performed on the predicted target genes of miR-142-5p. The gene ratio (x-axis) represents the enrichment ratio of miR-142-5p target genes (−log10-transformed), reflecting the degree of pathway enrichment (higher values indicate greater enrichment). The enriched KEGG pathways are shown on the y-axis. **(c)** Gene Ontology (GO) functional enrichment analysis was performed on the predicted target genes of miR-142-5p. The gene ratio (x-axis) represents the enrichment ratio of miR-142-5p target genes (−log10-transformed), while the enriched GO terms are shown on the y-axis. In panels **(b, c)**, the size of each dot represents the number of genes enriched in a given pathway (larger dots indicate a greater number of enriched target genes), while dot color varies according to the adjusted P-value range (red indicates higher P values, and blue indicates lower P values). **(d)** A miRNA–transcription factor (TF) regulatory Sankey diagram was generated. The dark red band on the left represents miR-142-5p, whereas the central bands represent different modes of TF–miRNA regulatory relationships. Yellow indicates transcription factors repressed by miRNAs, blue indicates transcription factors activated by miRNAs, and green indicates transcription factors regulated by miRNAs.

These 16 miRNAs were submitted to the comprehensive miRWalk database and the StarBase database. From the three integrated databases within miRWalk, target genes predicted by at least two databases were retained to ensure a sufficient number of candidate targets for subsequent functional enrichment annotation. In the StarBase database, predicted target genes were ranked according to AgoExpNum in descending order. The target genes screened from miRWalk were then intersected with the predicted results from StarBase, and miRNAs without predicted target genes were excluded. Ultimately, 13 miRNAs significantly upregulated in the T_SCM_ subset were identified, along with 170 corresponding target genes. A miRNA–target gene regulatory network was subsequently constructed using the visualization software Cytoscape (**Figure 3a**).

Gene Ontology (GO) and Kyoto Encyclopedia of Genes and Genomes (KEGG) functional enrichment analyzes of the predicted target genes were performed using the R package clusterProfiler. KEGG pathway analysis revealed that miR-142-5p–associated target genes were mainly enriched in the Ras signaling pathway and the Wnt signaling pathway (**Figure 3b**), whereas GO enrichment analysis indicated that these target genes were primarily associated with biological processes and molecular functions related to regulation of developmental growth, regulation of apoptotic signaling pathways, and T-cell differentiation (**Figure 3c**). Notably, both the Ras and Wnt signaling pathways play critical roles in the regulation of cell metabolism, apoptosis, and proliferation. Collectively, these findings suggest that miR-142-5p may influence T-cell differentiation and proliferation through modulation of its target gene network.

To further support the regulatory role of miR-142-5p in T-cell differentiation and proliferation, experimentally validated upstream transcription factors of miR-142-5p were retrieved from the TransmiR database, and a miRNA–transcription factor regulatory Sankey diagram was constructed. Transcription factors such as PPARG, TP53, SPI1, and SP1, identified in this analysis, have all been reported to participate in T-cell differentiation and development (**Figure 3d**). Together, these results further indicate a potential regulatory role for miR-142-5p in T-cell differentiation and proliferation.

### 3.3 PRKCB is a direct target gene of miR-142-5p in T cells

The target genes of miR-142-5p were predicted using the StarBase database. To further identify target genes associated with T-cell differentiation and development, reverse enrichment analysis was performed on the predicted miR-142-5p target genes using the online tool GeneTrail 3.2, with experimentally validated target genes excluded and a significance threshold of P < 0.05 applied. KEGG pathway analysis revealed that PRKCB and ANKRD17 were primarily enriched in the Wnt and Ras signaling pathways. Gene Ontology (GO) analysis further showed that PURA and CAMK2A were mainly enriched in biological processes and molecular functions including regulation of developmental growth, cell growth, RNA polymerase transcription regulator complex activity, and protein serine kinase activity. Collectively, these results suggest that PRKCB, PURA, ANKRD17, and CAMK2A are involved in the regulation of T-cell differentiation and function. Accordingly, these four genes were selected as potential miR-142-5p target genes for subsequent experimental validation.

**Figure 4:**
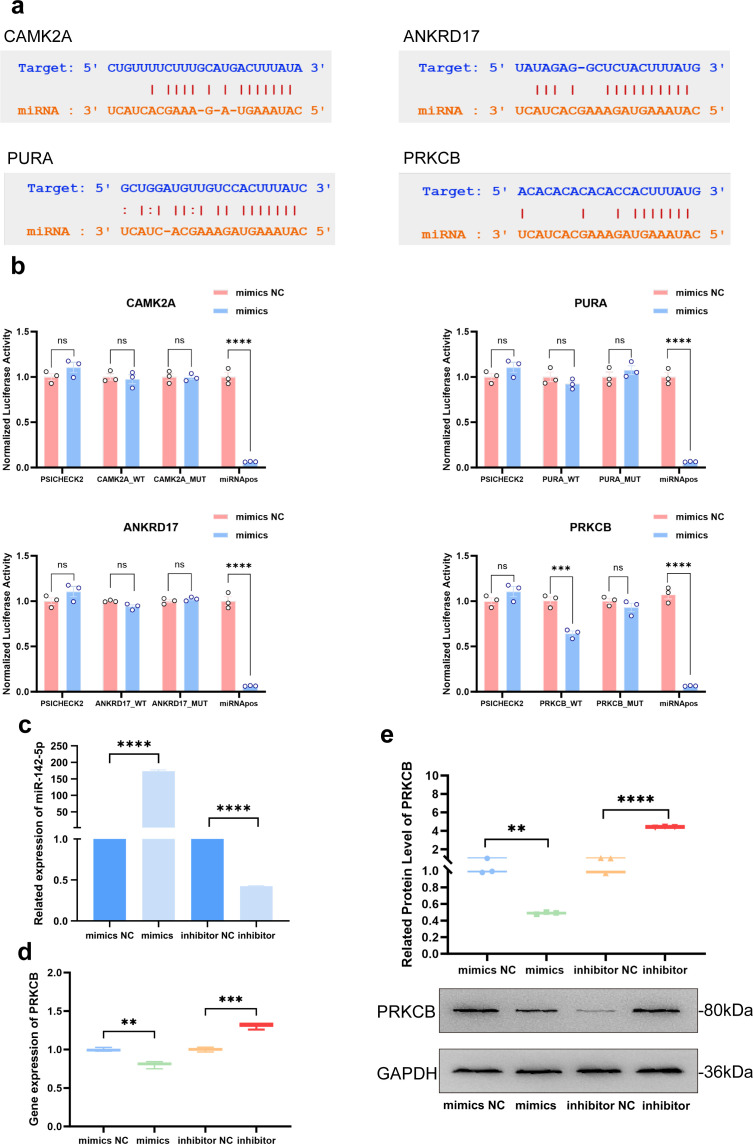
Validation of PRKCB as a direct target gene of miR-142-5p. **(a)** The binding sites of miR-142-5p within the 3′ untranslated regions (3′-UTRs) of PRKCB, PURA, ANKRD17, and CAMK2A were predicted using the StarBase database. **(b)** Dual-luciferase reporter assays performed in HEK293 cells demonstrated that miR-142-5p directly binds to the 3′-UTR of PRKCB, but not to the 3′-UTRs of PURA, ANKRD17, or CAMK2A. **(c)** Stimulated T cells were transfected with miR-142-5p mimics, mimics negative control (mimics NC), miR-142-5p inhibitors, or inhibitors negative control (inhibitors NC), respectively. The relative expression level of miR-142-5p was determined by quantitative real-time polymerase chain reaction (qPCR). **(d)** Following miR-142-5p mimics transfection, the mRNA expression level of PRKCB in T cells (normalized to GAPDH) was significantly reduced (P < 0.01), whereas transfection with miR-142-5p inhibitors resulted in a significant increase in PRKCB mRNA expression (P < 0.001). **(e)** Consistent with the mRNA results, PRKCB protein expression in T cells was significantly decreased following miR-142-5p overexpression (P < 0.01) and significantly increased after miR-142-5p inhibition (P < 0.0001). Data are presented as mean ± standard error of the mean (SEM), n = 3. Statistical significance was determined using Student’s t-test. ns, not significant; **P < 0.01, ***P < 0.001, ****P < 0.0001. T cells were activated by stimulation for 72 h prior to transfection with miR-142-5p mimics or inhibitors.

To further determine whether miR-142-5p could directly bind to these four potential genes and thereby regulate their expression, the putative miR-142-5p binding sites within the 3′ untranslated regions (3′-UTRs) of these genes were predicted using the StarBase database (**Figure 4a**). Wild-type luciferase reporter plasmids for each gene were then constructed, along with the corresponding mutant reporter plasmids in which the miR-142-5p binding sites were deleted.

Dual-luciferase reporter assays demonstrated that, compared with the mimics negative control (mimics NC) group, luciferase activity of the PRKCB wild-type reporter was significantly reduced following transfection with miR-142-5p mimics (P < 0.001). In contrast, no significant difference in luciferase activity was observed for the PRKCB mutant reporter between the miR-142-5p mimics and mimics NC groups. No statistically significant differences in luciferase activity were observed between the miR-142-5p mimics group and the mimics negative control (mimics NC) group following transfection with either the wild-type or mutant plasmids of CAMK2A, PURA, and ANKRD17 (**Figure 4b**).

These results indicate that miR-142-5p directly targets and binds to PRKCB, but does not exert a direct regulatory effect on CAMK2A, PURA, or ANKRD17.

Subsequently, the effects of miR-142-5p overexpression and inhibition on endogenous PRKCB expression in T cells were evaluated. T cells were transfected separately with miR-142-5p mimics, mimics NC, miR-142-5p inhibitors, or inhibitors negative control (inhibitors NC) using Lipofectamine 3000. Quantitative real-time polymerase chain reaction (qPCR) analysis confirmed that miR-142-5p expression was significantly increased in T cells following transfection with miR-142-5p mimics (P < 0.0001) (**Figure 4c**). At 48h post-transfection, changes in PRKCB mRNA and protein expression levels in T cells were assessed by quantitative real-time polymerase chain reaction and Western blotting (WB), respectively (**Figures 4d, e**). The results showed that both the mRNA and protein levels of PRKCB in T cells were significantly decreased after transfection with miR-142-5p mimics (P < 0.01), whereas PRKCB expression was significantly increased at both the mRNA and protein levels following miR-142-5p inhibition (P < 0.001).Collectively, these results confirm that transfection with miR-142-5p mimics leads to miR-142-5p overexpression in T cells and suppresses PRKCB expression by directly binding to its 3′-untranslated region (3′-UTR).

### 3.4 miR-142-5p suppresses the progressive differentiation of T cells

To elucidate the role of miR-142-5p in the progressive differentiation of T cells, activated T cells were stimulated for 72 h and subsequently transfected with miR-142-5p mimics, mimic negative control (mimics NC), miR-142-5p inhibitor, or inhibitor negative control (inhibitor NC) using Lipofectamine™ 3000 as the transfection reagent.

**Figure 5:**
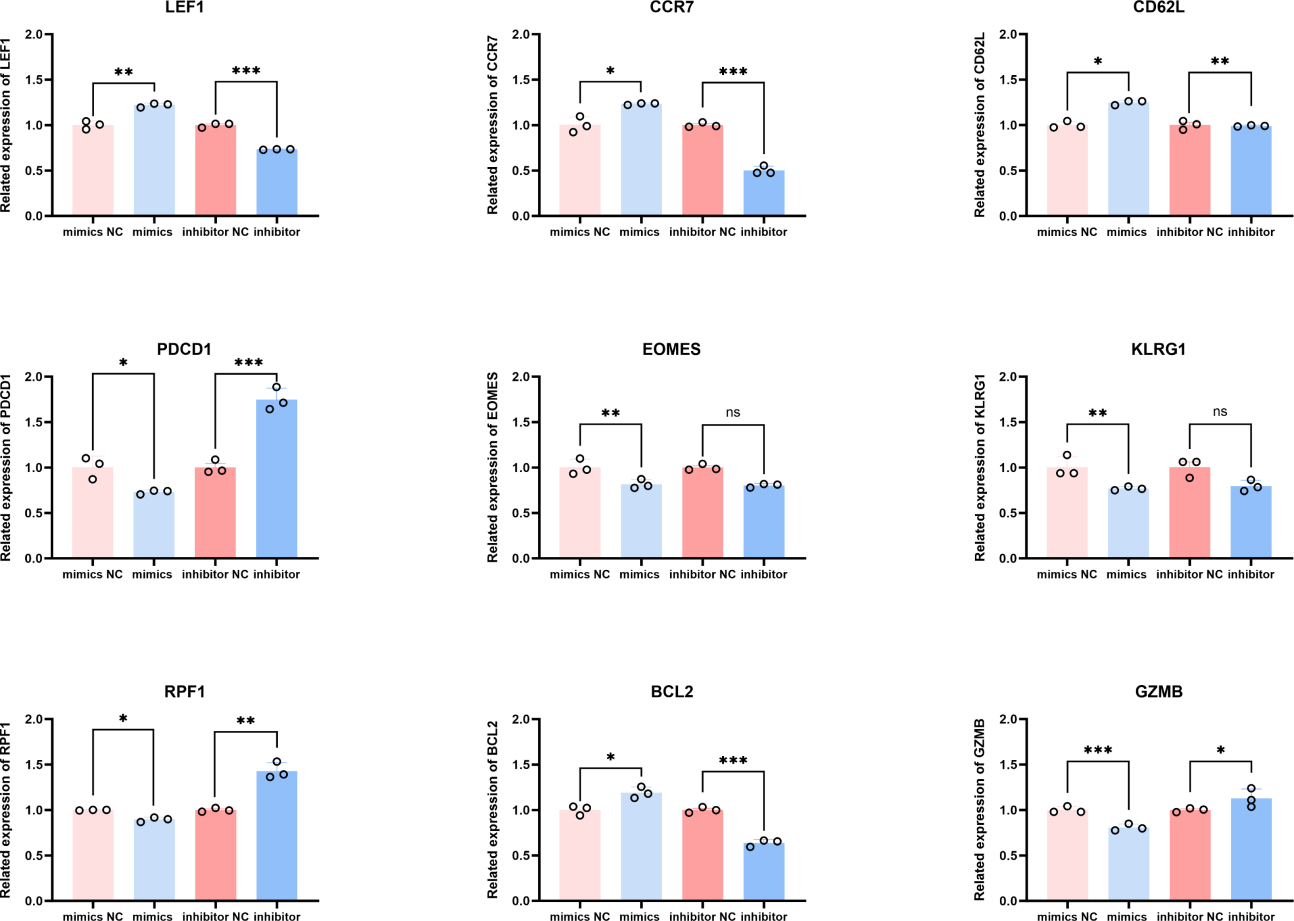
qPCR Analysis of effects of miR-142-5p on the expression of T-cell differentiation–related genes. Activated T cells were stimulated and subsequently transfected with miR-142-5p mimics, mimics negative control (mimics NC), miR-142-5p inhibitors, or inhibitors negative control (inhibitors NC). Total RNA was extracted using the Trizol method, and changes in the mRNA expression levels of early T-cell differentiation–associated genes (LEF1, CCR7, and CD62L), the anti-apoptotic gene BCL2, late differentiation–associated genes (KLRG1, EOMES, and PDCD1), and effector function–related genes (GZMB and PRF1) were quantified by quantitative real-time polymerase chain reaction (qPCR). Data are presented as mean ± standard error of the mean (SEM), n = 3. Statistical significance was determined using a paired Student’s t-test. *P < 0.05, **P < 0.01, ***P < 0.001.

We then examined the effect of miR-142-5p on the mRNA expression of genes associated with T-cell differentiation. As shown in **Figure 5**, At 48h post-transfection, the mRNA expression levels of nine representative genes were analyzed, including LEF1, CD62L, and CCR7 (early differentiation-associated genes), BCL2 (an anti-apoptotic gene), KLRG1, PDCD1, and EOMES (late differentiation-associated genes), as well as GZMB and PRF1 (effector function-related genes).

The qPCR results demonstrated that miR-142-5p overexpression significantly increased the expression of early differentiation-associated genes, including LEF1 (P < 0.01), CCR7 (P < 0.05), and CD62L (P < 0.05), as well as the anti-apoptotic gene BCL2 (P < 0.05). In contrast, the expression levels of late differentiation-associated genes, including PDCD1 (P < 0.05), GZMB (P < 0.001), and KLRG1 (P < 0.01), were significantly decreased following miR-142-5p overexpression.

Conversely, inhibition of miR-142-5p resulted in a significant reduction in BCL2 expression (P < 0.001), while the expression levels of late differentiation-associated genes, including PDCD1 (P < 0.001), GZMB (P < 0.05), and PRF1 (P < 0.01), were significantly increased. Collectively, these findings indicate that miR-142-5p overexpression shifts the transcriptional profile of T cells toward an early-differentiated phenotype.

**Figure 6:**
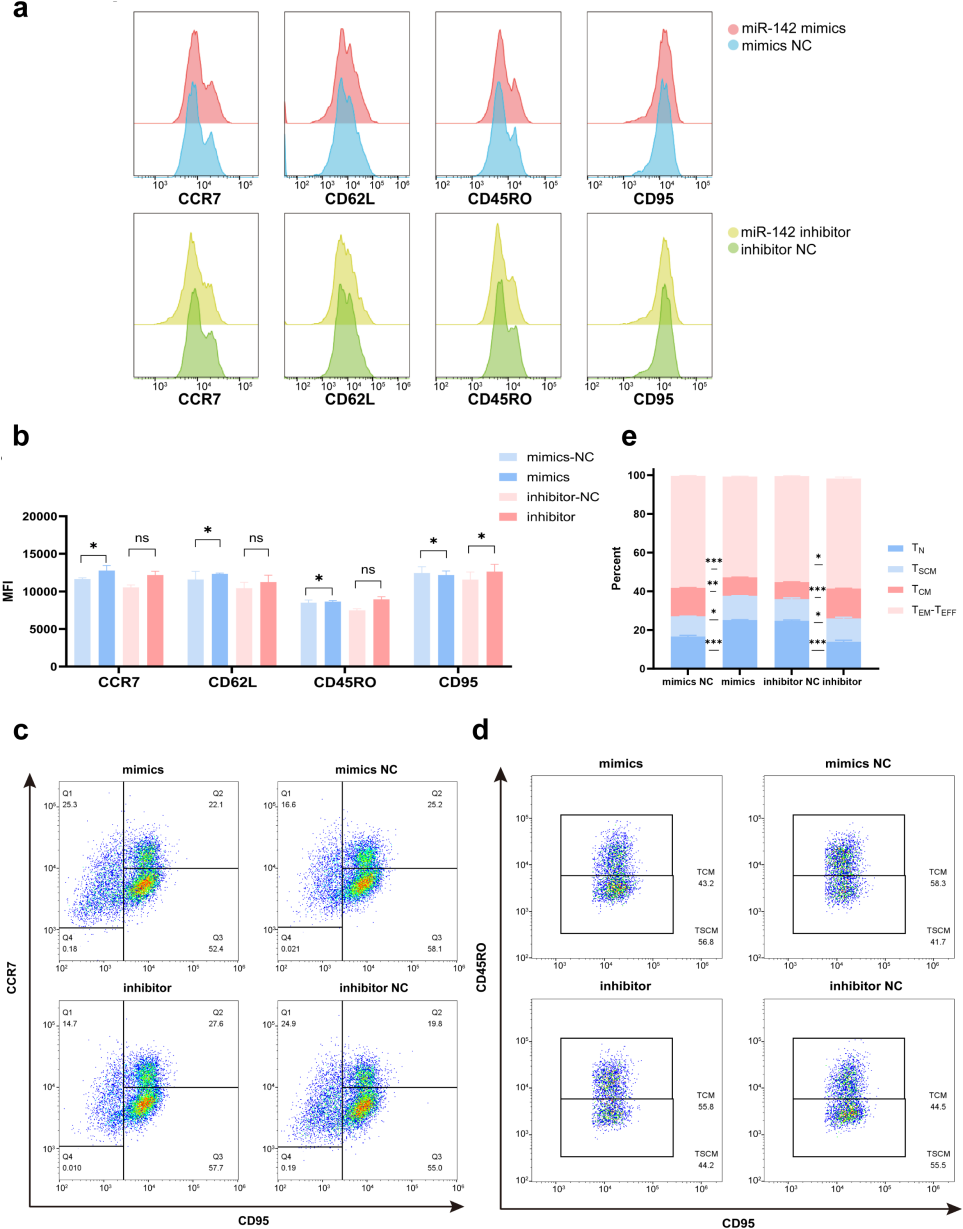
Effects of miR-142-5p on the differentiation phenotype of T cells. Stimulated T cells were transfected with miR-142-5p mimics, mimic negative control (mimic NC), miR-142-5p inhibitor, or inhibitor negative control (inhibitor NC). **(a)** The expression of CCR7, CD62L, CD45RO, and CD95 in CD3^+^ T cells were analyzed by flow cytometry. Data were processed using FlowJo software (version 10.9.0), and overlaid histograms were generated to display fluorescence intensity distributions of T cells stained with the indicated antibodies following transfection. **(b)** Statistical analysis of mean fluorescence intensity (MFI) corresponding to CCR7, CD62L, CD45RO, and CD95 expression was performed. **(c)** T cells(gated on CD3-postive T cells) were classified into differentiation-defined subsets based on CD95 and CCR7 expression: T_N_ (CD95^−^CCR7^+^), T_CM_ and T_SCM_ (CD95^+^CCR7^+^), T_EM_–T_EFF_ (CD95^+^CCR7^−^). **(d)** Cells within quadrant Q2 (CD95^+^CCR7^+^) were further analyzed and subdivided into T_SCM_ (CD95^+^CCR7^+^CD45RO^−^) and T_CM_ (CD95^+^CCR7^+^CD45RO^+^) subsets according to CD45RO expression. miR-142-5p overexpression significantly increased the proportion of the T_SCM_ subset, whereas miR-142-5p inhibition resulted in a significant decrease in this subset. **(e)** Based on the analyzes in panels **(c, d)**, the proportions of each T-cell subset were quantified and summarized in a stacked bar plot. Data are presented as mean ± standard error of the mean (SEM) (n = 3). Statistical significance was determined using a paired Student’s t-test; *P < 0.05, **P < 0.01, ***P < 0.001. T cells were activated by stimulation for 72 h prior to transfection.

We then validated the effects of miR-142-5p on the phenotypic profile of T cells. The expression levels of early differentiation markers (CCR7 and CD62L) and late differentiation markers (CD45RO and CD95) were assessed in CD3^+^-gated T cells by flow cytometry. As shown in **Figures 6a, b**, compared with the mimics negative control (mimics NC) group, miR-142-5p overexpression in T cells resulted in a significant increase in the mean fluorescence intensity (MFI) of CCR7 and CD62L (P < 0.05), accompanied by a significant decrease in the MFI of CD95 (P < 0.05). In contrast, inhibition of miR-142-5p in T cells led to a significant increase in CD95 MFI (P < 0.05).

To further evaluate the effect of miR-142-5p on T-cell differentiation, T cells at different stages of differentiation were classified into distinct subpopulations based on the expression of CD95 and CCR7: T_N_ (CD95^−^CCR7^+^), T_CM_ and T_SCM_ (CD95^+^CCR7^+^), and T_EM_–T_EFF_ (CD95^+^CCR7^−^). As shown in **Figure 6c**, compared with the mimics NC group, miR-142-5p overexpression significantly increased the proportion of the T_N_ subpopulation (P < 0.001) while significantly reducing the proportion of the TEM-T_EFF_ subpopulation (P < 0.001). Conversely, relative to the inhibitors negative control (inhibitors NC) group, miR-142-5p inhibition resulted in a significant reduction in the T_N_ subpopulation (P < 0.001), accompanied by a significant increase in the proportions of T_EM_-T_EFF_ subpopulation (P < 0.05).

For further analysis, cells located in quadrant Q2 (CD95^+^CCR7^+^) in **Figure 5c** were subdivided into T_SCM_ (CD95^+^CCR7^+^CD45RO^−^) and T_CM_ (CD95^+^CCR7^+^CD45RO^+^) subsets based on CD45RO expression (**Figure 6d**), as previously described. miR-142-5p overexpression led to a significant increase in the proportion of the T_SCM_ subset (P < 0.05), whereas miR-142-5p inhibition resulted in a significant decrease in this subset (P < 0.05).

The proportions of each T-cell subset were quantified by integrating all flow cytometry data (**Figure 6e**). The results demonstrated that miR-142-5p overexpression increased the proportion of early-differentiated T-cell subsets (T_N_ and T_SCM_), whereas miR-142-5p inhibition favored the accumulation of late-differentiated T-cell sets (T_CM_, T_EM_, T_EFF_). Collectively, these findings indicate that miR-142-5p suppresses progressive T-cell differentiation and contributes to the maintenance of an early differentiation state.

### 3.5 miR-142-5p promotes T-cell proliferation and suppresses T-cell effector function

**Figure 7:**
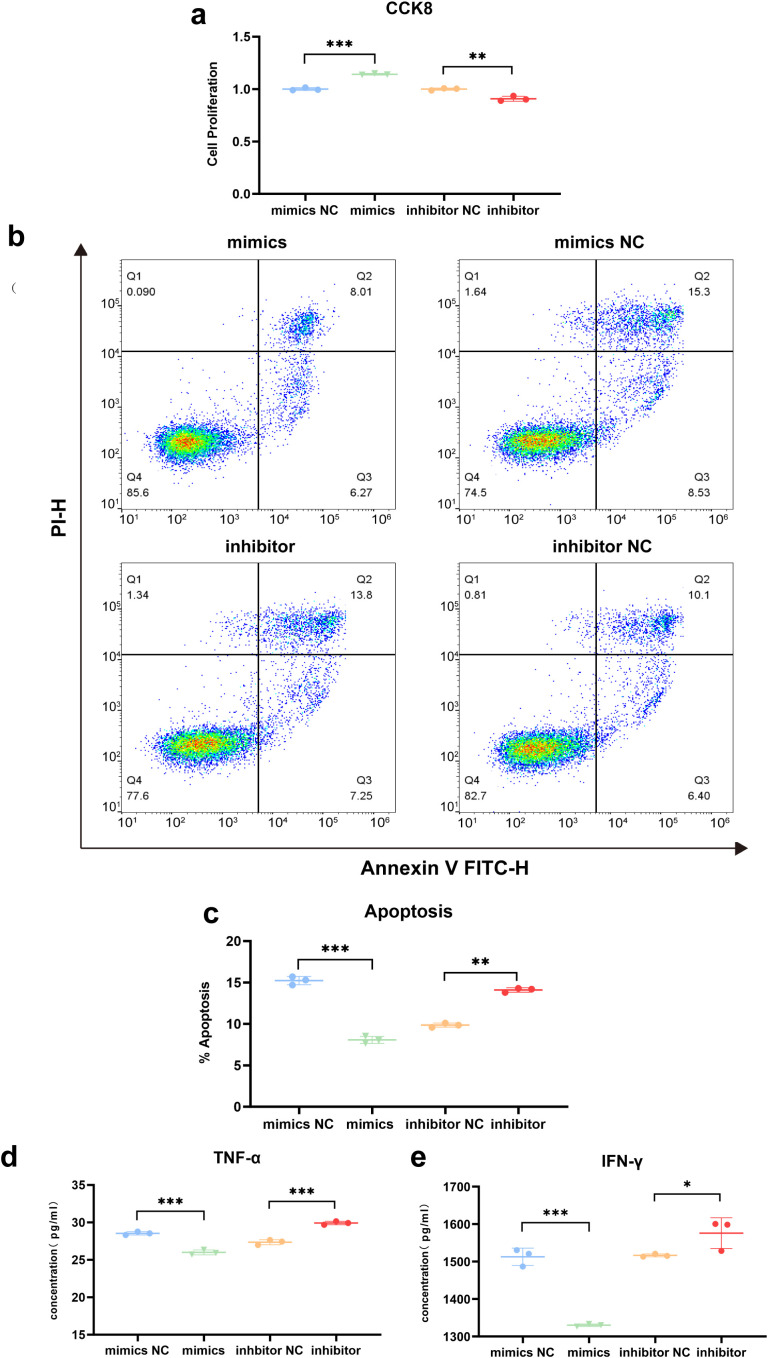
Effects of miR-142-5p on T-cell proliferation and effector function. Stimulated T cells were transfected with miR-142-5p mimics, mimic negative control (mimics NC), miR-142-5p inhibitor, or inhibitor negative control (inhibitor NC). **(a)** T-cell proliferation was assessed using the Cell Counting Kit-8 (CCK-8) assay. Compared with the mimics NC group, miR-142-5p overexpression significantly enhanced T-cell proliferation (P < 0.001), whereas miR-142-5p inhibition significantly reduced T-cell proliferation compared with the inhibitors NC group (P < 0.01). **(b)** The proportions of viable cells (Annexin V^−^/PI^−^), apoptotic cells (Annexin V^+^/PI^−^), and dead cells (Annexin V^+^/PI^+^) were analyzed by flow cytometry following Annexin V–FITC and propidium iodide (PI) staining. The x-axis represents FITC fluorescence intensity, and the y-axis represents PI fluorescence intensity. An increased proportion of viable cells was observed in the miR-142-5p overexpression group, whereas a decreased proportion of viable cells was observed in the miR-142-5p inhibition group. **(c)** Statistical analysis revealed that miR-142-5p overexpression significantly reduced the proportion of apoptotic and dead T cells (P < 0.001), whereas miR-142-5p inhibition significantly increased the proportion of these cells (P < 0.01). **(d, e)** The secretion of tumor necrosis factor-α (TNF-α) and interferon-γ (IFN-γ) by T cells was quantified using enzyme-linked immunosorbent assay (ELISA). TNF-α and IFN-γ secretion levels were significantly decreased in the miR-142-5p overexpression group (P < 0.001), whereas miR-142-5p inhibition resulted in significantly increased cytokine secretion (P < 0.05). Data are presented as mean ± standard error of the mean (SEM), n = 3. Statistical significance was determined using a paired Student’s t-test. P < 0.05, **P < 0.01, ***P < 0.001. T cells were activated by stimulation for 72 h prior to transfection.

As T cells undergo progressive differentiation, their proliferative capacity gradually declines, whereas their effector functions progressively increase. To assess the effect of miR-142-5p on T-cell proliferation, cell viability was measured at 48 h post-transfection using a Cell Counting Kit-8 (CCK-8) assay, and the relative proliferative activity of T cells in each group was calculated. The results showed that T-cell proliferative activity in the miR-142-5p mimics group was significantly higher than that in the mimics negative control (mimics NC) group (P < 0.001). Conversely, inhibition of miR-142-5p resulted in a significant decrease in T-cell proliferative activity compared with the inhibitors negative control (inhibitors NC) group (P < 0.01). These findings indicate that miR-142-5p positively regulates T-cell proliferation (**Figure 7a**).

At 6 days post-transfection, the proportions of apoptotic and dead T cells were assessed by flow cytometry using Annexin V–propidium iodide (PI) double staining. As shown in **Figures 7b, c**, the number of Annexin V–positive apoptotic and dead T cells in the miR-142-5p mimics group was significantly lower than that in the mimics negative control (mimics NC) group (P < 0.001). Conversely, inhibition of miR-142-5p expression using a specific inhibitor resulted in a significant increase in the number of Annexin V–positive apoptotic and dead T cells compared with the inhibitors negative control (inhibitors NC) group (P < 0.01). These results suggest that miR-142-5p enhances the anti-apoptotic capacity of T cells.

The secretion of pro-inflammatory cytokines, such as tumor necrosis factor-α (TNF-α) and interferon-γ (IFN-γ), represents a key effector function of T cells. At 6 days post-transfection, the effects of miR-142-5p on TNF-α and IFN-γ secretion were evaluated using enzyme-linked immunosorbent assay (ELISA) kits (**Figures 7d, e**). The results demonstrated that TNF-α and IFN-γ secretion levels were significantly decreased in T cells transfected with miR-142-5p mimics compared with the mimics negative control (mimics NC) group (P < 0.001). Conversely, miR-142-5p inhibition resulted in a significant increase in cytokine secretion compared with the inhibitors negative control (inhibitors NC) group (P < 0.05). Collectively, these findings indicate that miR-142-5p suppresses T-cell effector function while promoting T-cell survival and proliferation.

## 4 Discussion

In adoptive cellular therapy, exemplified by chimeric antigen receptor (CAR) T-cell therapy and T-cell receptor (TCR) T-cell therapy, accumulating clinical evidence indicates that the *in vivo* persistence and durability of antitumor efficacy of infused T cells are closely associated with their differentiation status. Compared with terminally differentiated T-cell subsets, such as effector T cells (T_EFF_) generated through *in vitro* activation and expansion, genetic modification of T cells at earlier stages of differentiation—including naïve T cells (T_N_), stem cell–like memory T cells (T_SCM_), and central memory T cells (T_CM_)—can significantly prolong *in vivo* survival and enhance proliferative capacity, thereby effectively extending antitumor activity *in vivo*.

To date, multiple strategies have been developed to increase the proportion of early-differentiated T cells, including the enrichment of early-differentiated T-cell subsets using flow cytometry or magnetic bead–based sorting techniques (13); the promotion of memory T-cell polarization and the attenuation of T-cell differentiation through optimization of *in vitro* culture conditions, such as the replacement of IL-2 with the cytokines IL-15 and IL-7 combined with low-dose anti-CD3/CD28 stimulation; the modulation of key differentiation-related signaling pathways (e.g., attenuation of excessive T-cell activation using kinase inhibitors); and the selection of 4-1BB rather than CD28 as the co-stimulatory domain in CAR construct design to favor the generation and maintenance of a memory phenotype (14).

In addition, reprogramming strategies have been explored to revert terminally differentiated T cells induced to an early phenotypic state, thereby enabling the generation of sufficient numbers of early-differentiated T cells. For instance, overexpression of early differentiation-associated transcription factors, such as TCF-1 and BATF3, not only enhances T-cell survival but also promotes conversion toward a memory phenotype. Moreover, the use of epigenetic modulators, including histone deacetylase inhibitors (15), has been shown to facilitate the maintenance of memory-associated characteristics in T cells. Metabolic reprogramming, achieved through the overexpression of FOXO1 (16) and the expression of an inhibition-resistant peroxisome proliferator–activated receptor gamma coactivator 1α (PGC-1α) (17), has also been reported to promote the generation of CAR-T cells with stem cell–like properties, thereby enhancing their antitumor activity *in vivo*.

In our previous studies, we explored strategies to reprogram terminally differentiated effector T cells (T_EFF_) by enforcing the expression of transcription factors characteristic of naïve T cells (T_N_) and stem cell–like memory T cells (T_SCM_). Through systematic screening, a combination of BCL6 and EOMES was identified, which was capable of promoting the dedifferentiation of T_EFF_ cells toward an early-differentiated T-cell phenotype.

In addition to transcription factors, microRNAs (miRNAs) have been shown to play a critical role in development and provide miRNA-mediated mechanisms that govern the fate of T cells. T-cell subsets at distinct stages of differentiation (e.g., T_N_, T_CM_, and T_EFF_) exhibit distinct miRNA expression profiles, which regulate T-cell fate determination, effector functions, proliferation, and apoptosis by controlling multiple mRNA targets through sequence-specific interactions. For example, miR-155 enhances STAT1/STAT5 signaling by inhibiting SOCS1 and SHIP1 (18), thereby promoting Th1 and Th17 differentiation while suppressing regulatory T-cell (Treg) formation (19); miR-146a negatively regulates the NF-κB pathway through a feedback mechanism by targeting TRAF6 and IRAK1 (20), thus restraining excessive T-cell activation; the miR-17~92 cluster activates the PI3K/AKT signaling pathway by downregulating PTEN and BIM (21), thereby promoting T-cell proliferation and differentiation; whereas miR-214, which is markedly upregulated upon T-cell activation, further enhances proliferative responses by inhibiting PTEN (22). Collectively, these studies have not only deepened our understanding of the molecular mechanisms underlying T-cell differentiation but have also identified potential therapeutic targets for the optimization of immunotherapies, including CAR-T cell therapy.

Building on our prior profiling of miRNA expression across T-cell subsets at four stages of progressive differentiation (T_N_, T_SCM_, T_CM,_ and T_EM_) using high-throughput sequencing, the present study performed pairwise differential expression analyzes of T_SCM_ vs. T_N_, T_SCM_ vs. T_CM_, and T_SCM_ vs. T_EM_. The intersection of miRNAs identified from these three comparisons was subsequently determined, yielding 32 miRNAs with significantly differential expression in T_SCM_ cells. After ranking the differentially expressed miRNAs according to P values, hsa-miR-142-5p was identified as the most significantly upregulated miRNA in T_SCM_ cells. The expression pattern of miR-142-5p across T-cell subsets was further validated by quantitative real-time polymerase chain reaction (qPCR), and the results were consistent with those obtained from high-throughput sequencing analysis.

Subsequently, the target genes of miR-142-5p were predicted, and functional enrichment analyzes were performed. KEGG pathway analysis showed that miR-142-5p target genes were mainly enriched in signaling pathways such as Ras and Wnt. Gene Ontology (GO) enrichment analysis revealed that these target genes were associated with several biological processes and molecular functions. These included regulation of developmental growth, regulation of apoptotic signaling pathways, and T-cell differentiation. Together, these findings suggest that miR-142-5p target genes are largely involved in transcriptional regulation and related regulatory processes. The Wnt signaling pathway plays a critical role in the generation and maintenance of memory T cells. Previous studies have demonstrated that in the absence of Wnt signaling, cytoplasmic β-catenin is phosphorylated by a destruction complex composed of APC, GSK-3β, and other component0073 (23), followed by ubiquitin-proteasome-mediated degradation. Through this pathway, dendritic cells regulate the differentiation of naïve T cells into memory T cells, and activation of Wnt signaling represents a key event during T-cell activation that influences subsequent differentiation processes.

In addition, a miRNA-target gene-transcription factor regulatory network for miR-142-5p was constructed, revealing that upstream transcription factors regulating miR-142-5p and its target genes are extensively involved in T-cell differentiation and development. Collectively, these results suggest that miR-142-5p may influence T-cell differentiation by modulating the activity of key signaling pathways, such as Wnt and Ras, through regulation of its target gene expression.

Following reverse functional enrichment analysis of the predicted miR-142-5p target genes, CAMK2A, PURA, ANKRD17, and PRKCB were preliminarily identified as genes involved in T-cell differentiation and development, suggesting that miR-142-5p may influence T-cell differentiation by regulating the expression of these four potential target genes. Dual-luciferase reporter assays demonstrated that miR-142-5p could directly bind to the 3′-untranslated region (3′-UTR) of PRKCB, whereas no direct binding was observed with the 3′-UTRs of the other three candidate target genes. Moreover, miR-142-5p overexpression in T cells resulted in a significant reduction in both PRKCB mRNA and protein expression levels, whereas inhibition of miR-142-5p led to a significant increase in PRKCB mRNA and protein expression. These results confirm that PRKCB is a direct target gene of miR-142-5p.

PRKCB encodes protein kinase C-β (PKCβ), a member of the protein kinase C family, and serves as a key downstream signaling molecule of phospholipase C-γ (24). Previous studies have established that PKCβ is primarily involved in B-cell receptor (BCR) signaling, where it represents the most highly expressed PKC member in B cells and is a key regulator of B-cell polarization and fate decision (25). In contrast, T-cell receptor (TCR) signaling in T cells is predominantly mediated by PKC (26). However, clinical studies have reported that mutations in the PKCβ gene are among the most frequent somatic mutations observed in adult T-cell leukemia and lymphoma (27), suggesting that PKCβ may influence T-cell differentiation and development by modulating NF-κB transcription, potentially through regulation of IKK phosphorylation and nuclear translocation of p65.

We further validated the role of miR-142-5p in T-cell differentiation by inducing its overexpression through transfection of T cells with miR-142-5p mimics and by suppressing its expression using miR-142-5p inhibitors. Quantitative real-time polymerase chain reaction analysis showed that miR-142-5p overexpression resulted in significantly increased mRNA levels of early T-cell differentiation-associated genes(LEF1, CD62L, and CCR7), as well as the anti-apoptotic gene BCL2, whereas the mRNA levels of late differentiation-associated genes (KLRG1, PDCD1, and EOMES) and effector function-associated genes (GZMB and PRF1) were significantly decreased. Consistently, flow cytometric analysis demonstrated that miR-142-5p overexpression led to increased expression of the early differentiation markers CCR7 and CD62L, accompanied by a significant reduction in the expression of the late differentiation marker CD95.

Further analysis of T-cell differentiation status revealed that miR-142-5p overexpression significantly increased the proportions of the T_N_ and T_SCM_ subsets, representing early-differentiated T-cell subpopulations, while concomitantly reducing the proportion of the T_EM_–T_EFF_ subset, indicative of late differentiation. In addition, we assessed the effects of miR-142-5p on T-cell proliferative capacity and effector function. The results showed that miR-142-5p overexpression enhanced T-cell proliferative activity, significantly decreased the proportion of apoptotic and dead cells, and suppressed the secretion of the effector cytokines TNF-α and IFN-γ. Collectively, these findings indicate that miR-142-5p suppresses progressive T-cell differentiation and contributes to the maintenance of an early-differentiated phenotypic state in T cells.

In summary, our findings indicate that miR-142-5p is highly expressed in the T_SCM_ subset following antigen-induced activation of naïve T cells and inhibits progressive T-cell differentiation by directly targeting PRKCB. As T cells undergo progressive differentiation toward the T_CM_-T_EM_-T_EFF_ phenotypes, miR-142-5p expression is gradually downregulated.

Direct evidence for miR-142-5p deficiency remains limited. However, indirect evidence supports its critical role in T-cell biology. Overexpression studies have shown that miR-142-5p modulates CD4+ T-cell differentiation by targeting SOCS1, shifting the balance toward Th1 cells (28). More importantly, Treg-specific deletion of miR-142 in mice results in lethal autoimmunity, with miR-142-5p identified as a key regulator of Treg suppressive function through PDE3b repression (29). These findings indicate that miR-142-5p is essential for maintaining T-cell homeostasis, and its absence likely impairs early T-cell differentiation, consistent with our observations.

To date, direct evidence for T-cell developmental or differentiation defects specifically caused by hsa-miR-142-5p. Previous studies have reported additional biological functions of miR-142. First, miR-142 suppresses tumor cell proliferation, invasion, and migration by targeting oncogenes such as CDK5 and SYDE2. Second, miR-142 plays a critical role in maintaining regulatory T-cell (Treg) homeostasis, proliferation, and suppressive function by modulating interferon-γ(IFN-γ) signaling, thereby preventing autoimmunity, and is therefore regarded as a core regulator of Treg cell function. By directly targeting multiple IFN-γ-related genes, including Hif1a and Ifnγr2, miR-142 attenuates IFN-γ signaling in Treg cells, functioning as a “molecular brake.” Deletion of miR-142 in Treg cells results in reduced Treg cell numbers and impaired suppressive capacity, accompanied by aberrant activation of effector T cells and excessive IFN-γ production, ultimately leading to fatal systemic autoimmune disease. Third, in non-small cell lung cancer, miR-142-5p expression has been reported to positively correlate with sensitivity to PD-1 antibody therapy, suggesting that miR-142-5p may serve as a potential biomarker for predicting responsiveness to immune checkpoint blockade.

Therefore, overexpression of miR-142-5p in T cells is expected to suppress progressive T-cell differentiation, maintain an early-differentiated phenotypic state, and prolong *in vivo* persistence. In addition, miR-142-5p overexpression suppresses tumor cell proliferation and increases sensitivity to PD-1 therapy. These results point to its potential to enhance combination immunotherapies involving CAR-T cells and PD-1 blockade.

## Data Availability

The raw sequencing data have been deposited in the NCBI SRA and are publicly accessible via the BioProject accession PRJNA1310025.
