# Supplementary material for: miR-142-5p promotes TSCM differentiation and suppresses progressive T-cell maturation via targeting PRKCB
Source: Front Immunol. 2026 May 18;17:1807053. doi: 10.3389/fimmu.2026.1807053 (PMC13222960; doi:10.3389/fimmu.2026.1807053)
Supplement: Supplementary file 1 [file DataSheet1.docx]

Supplementary Material

# Supplementary Data

## WB Original Image


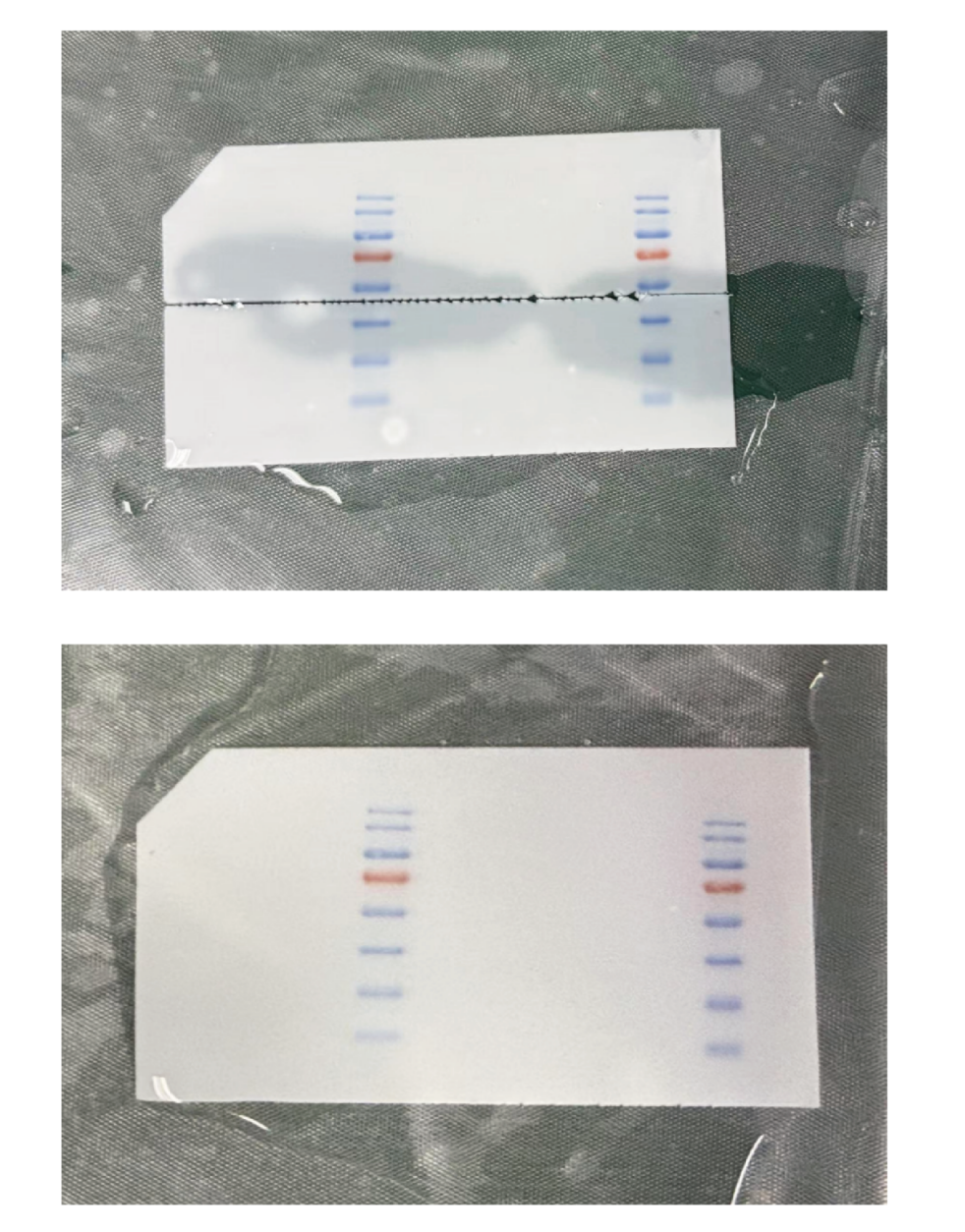


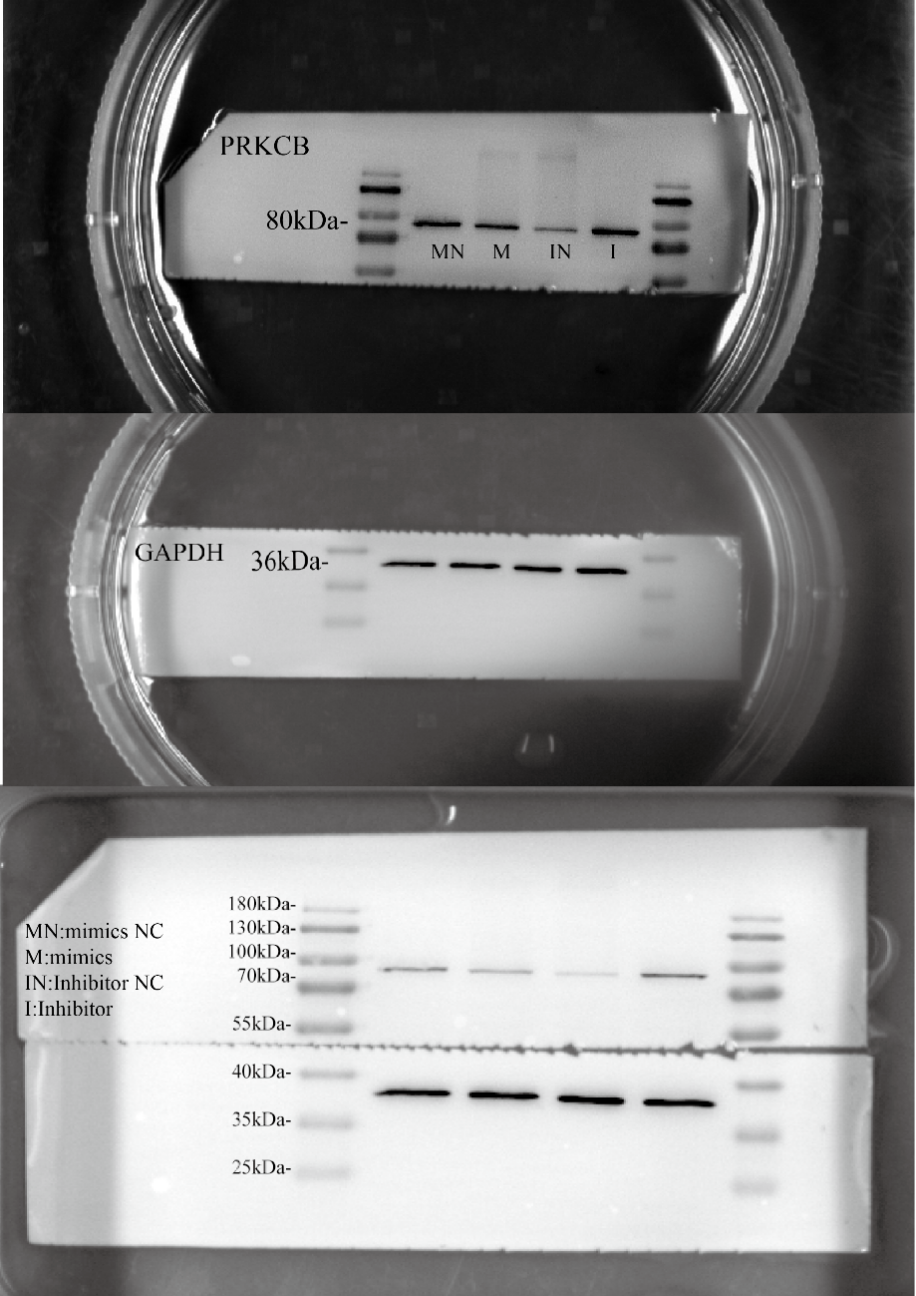


# Tables

## Supplementary Tables

**Table 1.**

| primers | Sequences |
| --- | --- |
| LEF1-Forward  LEF1-Reverse  CCR7-Forward  CCR7-Reverse  CD62L-Forward  CD62L-Reverse  PDCD1-Forward  PDCD1-Reverse  EOMES-Forward  EOMES-Reverse  KLRG1-Forward  KLRG1-Reverse  RPF1-Forward  RPF1-Reverse  BCL2-Forward  BCL2-Reverse  GZMB-Forward  GZMB-Reverse  LEF1-Forward  LEF1-Reverse  GAPDH-Forward  GAPDH-Reverse | CTCACATTAAGAAGCCTCTG  TACACTCAGCAACGACAT  TGGTCGTGGTCTTCATAG  TGCTACTGGTGATGTTGA  AACCGACTGCTGGACTTACC  AGATACTCAATTTCCGCCTTG  CTGTGTTCTCTGTGGACTA  AGACAATGGTGGCATACT  GATAGTCTTACAATCCTTACACAA  GCTCATTCAAGTCCTCCA  GACTATGGACCACAGCAA  TAGCAGCACACTCAGAAG  CTGTGAGGAGAAGAAGAAGA  TCGTTAATGGAGGTGTGAT  TGTGGATGACTGAGTACC  GAGACAGCCAGGAGAAAT  CCTGATACGAGACGACTT  TTCTTAGGATTATAGGCTGGAT  CTCACATTAAGAAGCCTCTG  TACACTCAGCAACGACAT  GGGAAACTGTGGCGTGAT  TCAGTGTAGCCCAGGATGC |
